# Resilience and Anxiety Among Healthcare Workers During the Spread of the SARS-CoV-2 Delta Variant: A Moderated Mediation Model

**DOI:** 10.3389/fpsyt.2022.804538

**Published:** 2022-02-16

**Authors:** Ying Liu, Tianya Hou, Hongjuan Gu, Jing Wen, Xiaoqin Shao, Yawei Xie, Wenxi Deng, Wei Dong

**Affiliations:** ^1^Faculty of Psychology, Navel Medical University, Shanghai, China; ^2^Department of Nursing, Hunan Provincial Crops Hospital, Chinese People's Armed Police Force, Changsha, China

**Keywords:** resilience, anxiety, general self-efficacy, positive coping style, coronavirus disease 2019 (COVID-19), healthcare workers

## Abstract

**Introduction:**

The B.1.617.2 (Delta) variant of SARS-COV-2 has caused a surge in COVID-19 cases worldwide, placing a great burden on the health care system under the zero-tolerance epidemic prevention policy in China. The present study aimed to investigate the prevalence of anxiety among health care workers during the spread of the SARS-CoV-2 Delta variant, and to discuss the mediating role of positive coping style between resilience and anxiety, and the moderating role of general self-efficacy.

**Method:**

Connor-Davidson Resilience scale (CD-RISC), Generalized Anxiety Disorder Scale (GAD-7), General Self-efficacy Scale (GSES) and Simplified Coping Style Questionnaire (SCSQ) were used in this cross-sectional study among 390 healthcare workers in Jiangsu Province, China. Mackinnon's four-step procedure was applied to test the mediation effect, and Hayes PROCESS macro was conducted to examine the moderated mediation model.

**Results:**

The prevalence of anxiety among Chinese healthcare workers during the spread of the SARS-CoV-2 Delta variant was 41.8%. Male, unmarried, childless and younger subjects reported higher levels of anxiety. Positive coping partially mediated the effect of resilience on anxiety among healthcare workers and the indirect effect was stronger with the increase of general self-efficacy.

**Conclusions:**

Anxiety was prevalent among healthcare workers during the spread of SARS-CoV-2 Delta variant. This research sheds new light on the potential mechanism underlying the association between resilience and anxiety and provides new insight into the prevention of anxiety among healthcare workers during the spread of the SARS-CoV-2 Delta variant.

## Introduction

The outbreak of the Coronavirus Disease 2019 (COVID-19) as a public health emergency with international concern (1) had an unprecedented impact on the daily life of people all over the world, causing approximately 4.5 million deaths and 216 million infections worldwide (2). Also, the continuously mutating severe acute respiratory syndrome coronavirus 2 (SARS-CoV-2) strain posed a major challenge to the health care systems.

Although the spread of COVID-19 in China has been controlled to a certain extent, the risk of being infected has not subsided (3). Moreover, the B.1.617.2 (delta) variant has delivered a huge shock even to countries that have been mass vaccinated, because of its higher load and faster spread than SARS-CoV-2 (4). Therefore, the first local case in May (5), 1 month after the previous outbreak, has caused a considerable degree of panic (such as anxiety) in China. To control the spread of the outbreak, patients need to be quickly identified and isolated by scaling up nucleic acid tests, which places a huge burden on the healthcare system. It can be inferred that the work efficiency and quality of healthcare workers have become the key to control the epidemic. However, the fear of being infected or bringing the virus to family, lack of knowledge about the Coronavirus, high levels of work stress and workload and inadequate psychological support during the COVID-19 pandemic have made healthcare workers more vulnerable to develop psychological problems than other groups (6–8). A great many of studies conducted early during the outbreak showed a high level of depression, anxiety and insomnia among healthcare workers (9, 10), suggesting that greater attention should be paid to the mental health of healthcare workers. Among these symptoms, anxiety as the most prevalent mental disorders (11) is of particular concern to us because it can directly or indirectly cause cognitive deficits, reducing job performance by limiting working memory (12) or affecting cognitive flexibility and decision-making (13). Anxiety disorder is a mental health condition characterized by excessive fear, anxiety, or avoidance of perceived threats to the external environment or internal as well as the actual response is not equal to the actual risk (14). It is one of the most predominant mental disorders in the general population (11). A large web-based cross-sectional study conducted across China reported that the overall prevalence of general anxiety disorder (GAD) during the COVID-19 epidemic was 35.1%, and healthcare workers were at a higher risk of mental illnesses (15). Numbers of recent studies in the field of positive psychology have focused on anxiety disorders (16–18), and psychological resilience as an important component of positive psychology is also suggested to have a protective effect on anxiety (16).

Resilience refers to the capacity that allows people to successfully adapt and face adversity, traumatic and stressful events (19). The negative association between resilience and anxiety has been confirmed by multiple studies (20, 21). Moreover, an observational longitudinal cohort study conducted in individuals with multiple sclerosis over 12 months confirmed a significant longitudinal relationship between resilience and anxiety (22). When confronted with stressful life events, individuals with higher levels of resilience were less likely to experience anxiety and depression (23). A recent study reported the protective role of resilience components against mental problems including anxious symptoms among Italian healthcare workers during COVID-19 pandemic (24). Thus, we speculate that resilience may have a protective effect on anxiety of Chinese healthcare workers during the COVID-19 pandemic.

### The Mediating Role of Coping Style

Despite the associations between resilience and anxiety having been well established, the underlying mechanisms behind this association have not been fully explained. Specifically, whether the association between resilience and anxiety among healthcare workers during the spread of the SARS-CoV-2 Delta variant is mediated by coping style has not been tested.

Coping is the cognitive and behavioral effort of individuals to consciously manage external or internal changes (25), which can be divided into two types according to the ways of coping with problems: positive coping and negative coping (26). Positive coping refers to solving problems in a direct and rational way such as focusing on the positive and changing behaviors to solve problems and seeking social support (27), while negative coping refers to dealing with problems through avoidance, withdrawal and denial (28). However, extant literature has already documented that positive coping is the dominant coping style among medical students or people facing COVID-19 (29–31), whereas multitudes of studies only investigated the impact of negative coping style (28, 32, 33). Therefore, the present study would focus on the effects of positive coping, and we will consider positive coping in our study.

The association between resilience and coping styles has attracted much attention. Similarly, a study conducted among Chinese soldiers found that resilience was a positive predictor of positive coping (34). A recent study reported the positive association between resilience and positive coping based on a sample of healthcare workers during the outbreak (21). According to the transactional stress model, coping plays an important role when individuals face adversity, and rapid response to stress is beneficial to prevent the generation of psychological disorders (35). Many empirical studies have reached the consensus that positive coping was a protective factor for anxiety, while negative coping may exacerbate this symptom (36, 37). In addition, a longitudinal study conducted in the United States showed that a lower level of positive coping among patients with post myocardial infarction was associated with a higher level of anxiety (38). Moreover, several studies provided robust evidence for the negative association between positive coping and anxiety among healthcare workers (39, 40). Therefore, it could be speculated that positive coping mediated the association between resilience and anxiety among healthcare workers.

To date, the association between resilience, coping style and anxiety has been widely investigated (41–43). However, some of these studies focused on patients rather than medical staff, and others used coping style as an independent variable or resilience as a moderator. To the best of our knowledge, the association between resilience and anxiety *via* positive coping among health care workers during the COVID-19 outbreak has not been studied.

### The Moderating Effect of General Self-Efficacy

Although resilience may affect anxiety indirectly through positive coping, not all people who are more inclined to use positive coping reported a lower level of anxiety since some studies reported no association between positive coping and anxiety (44). Therefore, it is necessary to explore the influencing factors of the association between positive coping and anxiety. Self-efficacy was defined as a belief in one's ability to handle complex or new tasks and cope with adversity, which exerted an impact on how people feel, think and behave (45). General self-efficacy is a generalized sense of self-efficacy, which refers to global confidence in one's ability to cope with a variety of different demands or new situations (46). In the light of the Integrative Conceptual Framework of coping process, individual's self-efficacy as a personal characteristic can interact with coping styles or coping skills to influence personal health and well-being (47), indicating the effect of coping skills on health differs at different levels of self-efficacy. Previous literature presented the interaction effect of coping style and self-efficacy on the treatment outcome among problem drinkers (48), suggesting the influence of coping style on health outcomes is not the same at different levels of self-efficacy. Brands and colleagues explored the influence of self-efficacy and negative coping on quality of life and found self-efficacy moderated the impact of emotion-oriented coping on health outcome. Specifically, the effect of negative coping on health outcome was attenuated with the increase of self-efficacy (49). Hence, we speculate that self-efficacy may moderate the effect of positive coping on anxiety among healthcare workers during the crisis.

### The Present Study

**Figure 1:**
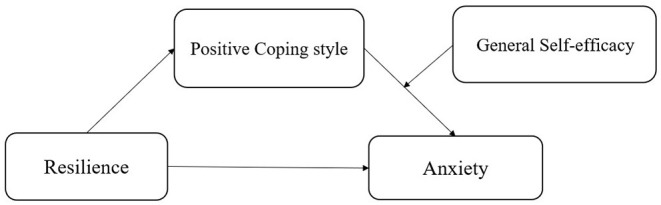
The schematic model of proposed moderated mediation model.

The purpose of this study is to investigate the prevalence of anxiety among health care workers during the spread of the SARS-CoV-2 Delta variant, and to discuss the mediating role of positive coping style between resilience and anxiety, and the moderating role of general self-efficacy. Taken together, our study proposes a moderated mediation model that general self-efficacy moderates the indirect effect (positive coping–anxiety) of resilience on anxiety through positive coping style (see Figure 1) among healthcare workers during the COVID-19 pandemic.

## Methods

### Participants and Procedures

The cross-sectional study was performed between May 14 and 25, 2021, during the spread of the SARS-CoV-2 Delta variant. A one-stage random cluster sampling technique was employed to recruit participants from a hospital in JiangSu Province. A total of 413 potential participants were contacted in the study. The inclusion criteria were as follows: (a) working in the hospital for at least 1 year, (b) no dyslexia or cognitive impairment, (c) age > 18 years. The exclusion criterion was set for respondents with psychiatric illnesses and those who did not respond seriously. Finally, 390 participants were included in the analysis, resulting in a valid response rate of 96.13% (390/413). The research project obtained an ethical approval from Suzhou Science & Technology Town Hospital (IRB201912002RI) before it was launched. All data were collected by conducting a self-administered questionnaire online. Prior to the online survey, informed consent online was given by all participants. Also, all participants were assured that their responses would be anonymous and confidential and that they were free to withdraw at any time without penalty.

### Measures

#### Demographics Characteristics of Participants

Demographic information in this study included gender, age, educational level, professional title, marital status, and children situation. Age was divided into two groups (younger group and middle-age group). Educational level was categorized into two groups (college or lower, Master degree or above). Professional title was coded as a binary variable (junior title or no title, intermediate job title and senior title). Marital status was divided into married and unmarried (single, divorced, and widowed). Children situation was categorized into no child and having at least 1 child.

#### Measurement of Resilience

The Chinese version of Connor-Davidson Resilience scale (CD-RISC) (50) is a 25-item generic resilience instrument with three subscales: tenacity, strength, and optimism. Items were scored on a 5-point Likert scale ranging from 0 (never) to 4 (very often). A total score is calculated as the sum of all questions and ranged from 0 to 100, and the higher the score is, the higher the level of resilience is. The scale has been demonstrated good internal and external validity and widely employed in Chinese healthcare workers (51). In this study, the Cronbach's α coefficient of the scale was 0.968.

#### Measurement of Anxiety

The 7-item Generalized Anxiety Disorder Scale (GAD-7) was used to measured anxiety of patients during the last 2 weeks (52). The variables were scored on a four-point Likert scale with 0 indicating never,1 indicating several days, 2 indicating more than half the days, and 3 representing nearly every day. The total score was calculated as the sum of all items, with a total range of 0 to 21. The higher the total score is, the more severe the anxiety is. The cut-off point for identifying the symptoms of anxiety was 7 (53). The scale has been widely used in anxiety-related research and has high construct validity and reliability in Chinese population (54, 55). In the present study, the Cronbach's α coefficient of the scale was 0.966.

#### Measurement of Positive Coping Style

Positive coping was measured by the positive coping subscale of Simplified Coping Style Questionnaire (SCSQ) (56). The SCSQ was an instrument widely used in China to reflect positive and negative responses when encountering stress (56). The positive coping subscale consists of 12 items (e.g., release through work, study or some other activities). The SCSQ was scored on a 4-point Likert scale ranging from 0 (do not take) to 3(often take). The positive coping subscale was calculated as the sum of all items. The total score of items represents the likelihood that the individual will adopt the corresponding coping style, with higher scores reflecting stronger coping style preferences (57). The scale has presented excellent psychometric properties and been widely used among healthcare workers (56), and the Cronbach's alpha of positive coping in this study was 0.947.

#### General Self-Efficacy

To assess general self-efficacy, we used the Chinese version of 10-item General Self-efficacy Scale (GSES) (58). Items were rated on a 4-pointed Likert scale ranging from 1 (not true at all) to 4 (exactly true), with a total score ranging from 10 to 40. Higher scores indicated higher levels of general self-efficacy. The scale has been found to have good reliability and validity among Chinese healthcare workers (58, 59). In this study, the Cronbach alpha coefficient for GSES was 0.954.

### Statistical Analysis

SPSS 22.0 (IBM Corporation) was used for statistical analysis in this study. First of all, we conducted Harman single factor test to examine common method bias. Common method bias as a well-documented phenomenon observed in research based on self-reported measures is caused by the fact that the constructs are measured by the same methods (e.g., multiple-item scales in the same questionnaire), which might result in spurious effects because of measurement instruments (60). Then, an analysis of descriptive statistics was conducted to illustrate the demographic and other selected characteristics of the respondents. Independent *t*-test and one-way analysis of variance (ANOVA) were used to compare group differences in Anxiety. Secondly, Pearson correlation test was utilized to evaluate the bivariate correlations between interested variables. Thirdly, MacKinnon's four-step method (61) was applied to test the mediation effect in our research and four criteria need to be satisfied: (1) a significant association between the independent variable (resilience) and the dependent variable (anxiety); (2) a significant association between the independent variable (resilience) and the mediator (positive coping style); (3) a significant association between the mediator (positive coping style) and the dependent variable (anxiety) after controlling for the independent variable (resilience); (4) a significant coefficient for the indirect association between the independent variable (resilience) and the dependent variable (anxiety) *via* mediator (positive coping style). To examine the last condition, the bias-corrected percentile bootstrap method was used, obtaining the bias-corrected 95% confidence intervals with 5,000 bootstrapping iterations. If the interval range of 95% CI value does not contain 0, indicating that the mediating effect is significant. The mediation effect was analyzed by PROCESS version 3.0 macro for SPSS (Model 4), which is a free mediation and moderation software package published by Preacher and Hayes. Finally, the PROCESS macro (Model 14) was used to examine the moderated mediation effects. According to the foregoing, the effects were established if 95% bias-corrected bootstrap CI of the interaction does not contain zero. Then, Johnson-Neyman technique (62) was employed to plot the conditional effects and confidence bands at different values of general self-efficacy. In addition, gender, age, educational level, years of working, professional title, marital status, and children situation were entered into models as covariates and all continuous variables were standardized. In all data analysis, *p*-values of 0.05 or less (*p* < 0.05) were considered as statistical significance.

## Results

### Common Method Bias Test

In this research, we used self-report approach to collect data, which may lead to common method bias problem (63). The Harman single factor test was employed to test common method bias (64). The KMO value was 0.95 (p < 0.001), indicating that the data in this study were suitable for exploratory factor analysis. After exploratory factor analysis, we found that the factors of eigenvalues >1 was 8 and the interpretation rate of the first factor was 37.42%, lower than the reference value of 40%. Therefore, the results showed that there was no serious common method bias problem in this research.

### Demographic Characteristics and Anxiety

The sociodemographic characteristics and intergroup comparison of anxiety were displayed in Table 1. Among the 390 valid samples, the average age was 29.78 (±5.35) years old, and the average years of working was 7.84 (±5.73) years. Most of the participants were female [343(87.95%)], married [256(65.64%)], junior title [267 (68.46%)], aged below 30 years [249 (63.85%)], had at least one child [212 (54.36%)], had an educational level of college or lower.

**Table 1:** Demographic characteristics of respondents (*N* = 390) and group comparisons on anxiety.

	**Respondents**		**Anxiety Scores**		***F***/***t***	***p*** **-value**
	***n***	**%**	***M***	**SD**		
Gender					7.51	0.01
Male	47	12.05	6.72	5.39		
Female	343	87.95	4.76	4.49		
Marital status					5.59	0.02
Unmarried	134	34.36	5.76	4.86		
Married	256	65.64	4.60	4.48		
Children situation					6.60	0.01
No child	178	45.64	5.65	4.77		
One child or more	212	54.36	4.45	4.47		
Professional title					2.53	0.11
Junior title	267	68.46	5.25	4.71		
Intermediate job title	123	31.54	4.45	4.46		
and senior title						
Age (29.78 ± 5.35)					4.46	0.04
Younger group (≤ 30)	249	63.85	5.37	4.83		
Middle-aged group (>30)	141	36.15	4.34	4.22		
Educational level					1.71	0.19
College or lower	360	92.31	5.09	4.70		
Master degree or above	30	7.69	3.93	3.83		

The prevalence of anxiety among healthcare workers was 41.8%. There were no significant differences in the prevalence of anxiety among participants with different professional title and educational level. Of the total sample, males had higher levels of anxiety than females (*F* = 7.51, *P* < 0.05). Unmarried (*F* = 5.59, *P* < 0.05), childless (*F* = 6.60, *P* < 0.05) and younger group subjects (*F* = 4.46, *P* < 0.05) reported a higher level of anxiety.

### Mean, Standard Deviations (SD), and Bivariate Correlation of all Study Variables

Table 2 shows the Pearson correlation coefficient among variables. Resilience was positively associated with positive coping style (*r* = 0.70, *P* < 0.001) and general self-efficacy (*r* = 0.53, *P* < 0.001). Also, positive coping was positively related to general self-efficacy (*r* = 0.46, *P* < 0.001). Besides, resilience (*r* = −0.22, *P* < 0.001) and positive coping style (*r* = −0.32, *P* < 0.001) were negatively correlated with anxiety. However, general self-efficacy was not significantly related to anxiety (*P* > 0.05).

**Table 2:** Pearson's correlation among resilience, self-efficacy, coping style and anxiety (*N* = 390).

	**Mean (SD)**	**1**	**2**	**3**
1. Resilience (CD-RISC)	63.28 (14.83)	1.00		
2. Positive coping style (SCSQ)	24.65 (6.07)	0.70^***^		
3. General self-efficacy (GSES)	25.96 (5.90)	0.53^***^	0.46^***^	
4. Anxiety (GAD-7)	5.00 (4.64)	−0.22^***^	−0.32^***^	−0.07

*** *P < 0.001*.

### Mediating Effect of Positive Coping Style

After finding an internal links among resilience, anxiety, and positive coping style, the research examined the potential mediating role of positive coping style between resilience and anxiety. We used Mackinnon's four-step procedure to examine the mediation effect (see Table 3), which follows: above all, resilience was significantly correlated with anxiety (β = −0.250, *P* < 0.001) (see Model 1). Secondly, resilience was significantly associated with positive coping style (β = 0.742, *P* < 0.001) (see Model 2). Next, positive coping style was significantly related to anxiety when controlling for resilience (β =-0.286, *P* < 0.001) (see Model 3). Finally, the indirect effect of resilience on anxiety *via* positive coping style was significant (ab =-0.213, SE = 0.050, 95% CI = [−0.312, −0.117]). The mediation effect of positive coping style accounted for 85.31% of the total effect. The 95% CI did not contain zero, suggesting the indirect association between resilience and anxiety *via* positive coping style. In conclusion, mediation effect met all four conditions and positive coping style mediated the relation between resilience and anxiety among healthcare workers during the outbreak of COVID-19.

**Table 3:** Mediation analysis (*N* = 390).

	**Model 1 (Anxiety)**		**Model 2 (Positive coping)**		**Model 3 (Anxiety)**			**Indirect effect of positive coping style**			
	**β**	***t***	**β**	***t***	**β**	***t***		**Indirect effect**	**SE**	**LLCI**	**ULCI**
Resilience	−0.250^***^	−4.889	0.742^***^	18.906	−0.037	−0.529	Positive coping	−0.213	0.050	−0.311	−0.117
Positive coping					−0.286^***^	−4.415					
*R*^2^_adj_	0.099^***^		0.490^***^		0.142^***^						
*F*(d*f*)	5.966		52.452		7.909						
*P*	0.001		0.001		0.001						

*All models are adjusted for gender, marital status, age, children situation, educational level, and professional title*.

*** *P < 0.001*.

### Moderating Effect of Self-Efficacy

The study hypothesized that general self-efficacy might moderate the indirect effect (the second stage of the mediation pathway: positive coping-anxiety) of resilience on anxiety. The results of conditional process analysis in Table 4 showed the interaction of positive coping style and general self-efficacy had a significant effect on anxiety (β = −0.183, *P* < 0.001), indicating the association between positive coping style and anxiety was moderated by general self-efficacy. Therefore, the moderated mediation effect was established since the indirect pathway was moderated by general self-efficacy (65).

**Table 4:** Conditional process analysis (*N* = 390).

	**β**	**SE**	**LLCI**	**ULCI**
**Dependent variable model (outcome: anxiety)**				
Resilience	−0.121	0.071	−0.261	0.020
Positive coping style	−0.306^***^	0.064	−0.431	−0.181
Self-efficacy	0.179^**^	0.056	0.070	0.289
Positive coping style ^*^ Self-efficacy	−0.183^***^	0.038	−0.258	−0.109
	***β***	**Boot SE**	**Boot LLCI**	**Boot ULCI**
**Conditional indirect effect analysis**				
1 SD below the mean	−0.090	0.052	−0.195	0.013
Mean	−0.225	0.049	−0.325	−0.129
1 SD above the mean	−0.361	0.058	−0.478	−0.248
Index of moderated mediation	−0.136	0.025	−0.184	−0.085

*All models are adjusted for gender, marital status, age, children situation, educational level, and professional title*.

*** *P < 0.001*,

** *P < 0.01*.

**Figure 2:**
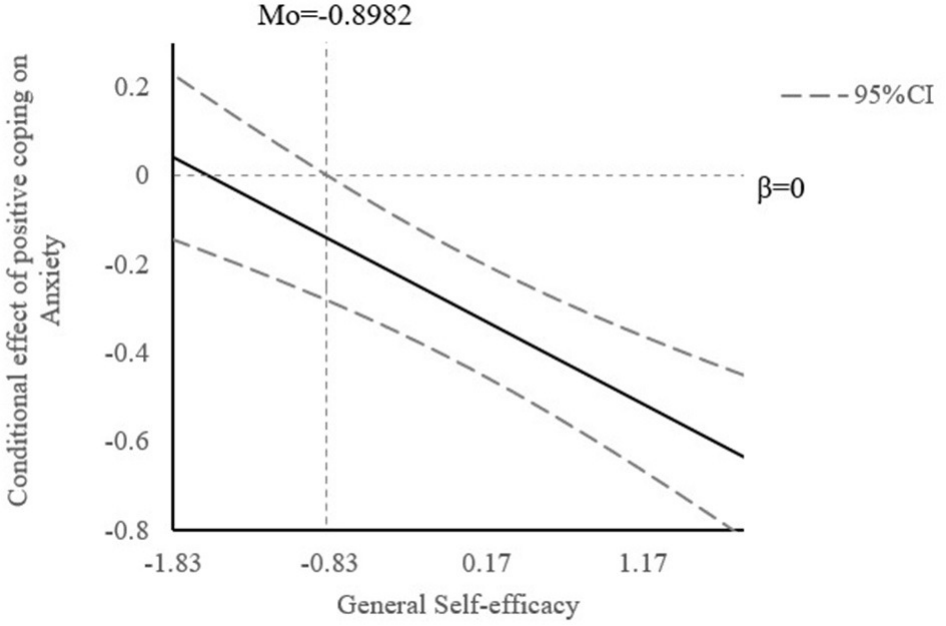
The conditional effect of positive coping on anxiety at the value of general self-efficacy.

The conditional indirect effect of resilience on anxiety *via* positive coping style at different values of general self-efficacy (1 SD below the mean, mean, and 1 SD above the mean) is also showed in Table 4. The indirect effect of positive coping style at 1 SD above the mean [β = −0.361, 95% CI (−0.478, −0.248)] was stronger than 1 SD below the mean [β = −0.090, 95% CI (−0.195, 0.013)]. As shown in Figure 2 by Johnson-Neyman technique (62), general self-efficacy would moderate the indirect effect of resilience on anxiety *via* positive coping when the standard scores of general self-efficacy were higher than −0.8982, in which the 95% CI did not contain zero.

## Discussion

This study aimed to assess the prevalence of anxiety symptoms among healthcare workers 20 months during the spread of the SARS-CoV-2 Delta variant, and to discuss the mediating role of positive coping style in the association of resilience with anxiety, and the moderating role of general self-efficacy. As far as we know, this is the first research to investigate the association between resilience and anxiety *via* positive coping and the moderating role of general self-efficacy.

The results showed that the overall prevalence of anxiety among Chinese healthcare workers during the spread of the SARS-CoV-2 Delta variant was 41.8%, which is higher than the prevalence of 35.1% reported in a large-scale epidemiological survey conducted among Chinese during the COVID-19 pandemic (15). This is also higher than the prevalence of 25% among healthcare workers during the peak period of COVID-19 reported in a meta-analysis (66). These suggest that, under the circumstance of the COVID-19 pandemic the constantly mutating virus, makes healthcare workers even more likely to be anxious in comparison to the peak of the epidemic.

The demographic variables showed that male subjects had higher levels of anxiety than females, which is inconsistent with previous findings (67–69). This might be explained by several reasons. First, different samples and questionnaires were used among these studies, which resulted in certain differences. In addition, the cluster sampling method adopted in this study resulted in a large difference in the number of men (only 47) and women. This could be attributed to the fact that most nursing staff were female (70). The results also presented that unmarried, childless, and younger subjects reported a higher level of anxiety, which is in line with some published findings (69, 71, 72). These results may be due to the fact that medical workers with these characteristics tend to undertake more workload and frontline duties. Also, their professional experience and decision-making authority are lower than those of senior medical staff (69, 73).

### The Mediating Role of Positive Coping

As expected, the results of MacKinnon's four-step method presented that the mediation effect accounted for 85.31% of the total effect, which indicated the effect of resilience on anxiety was largely through positive coping among healthcare workers during the spread of the SARS-CoV-2 Delta variant. This is consistent with the findings from previous literature (35, 74), which documented the mediating role of resilience in the association between resilience and health outcomes. Zhao et al. (35) found that resilience was correlated with positive coping, and coping style mediated the association between resilience and depressive symptoms. Chen (74) also proved that coping styles played a mediating role in the association between resilience and subjective well-being. The results could also be explained by the theory of psychological stress and coping developed by Lazarus et al., which claims that coping is a key mediator of stressful person-environment relations and their immediate and long-range outcomes (75). Therefore, appropriate coping styles play an important role in preventing individuals in stressful situations from developing short-term or long-term negative emotions. An individual with a higher level of resilience is more likely to develop positive coping strategies (76), which could further protect against anxiety disorders. Hence, positive coping, as a direct and rational way, could be a good mediator between the resilience and anxiety of medical staff under the COVID-19 pandemic, indicating resilience can have an impact on anxiety through positive coping.

### The Moderating Role of General Self-Efficacy

In the moderated mediation analysis, the coefficient of the interaction term between self-efficacy and coping is significant, suggesting the moderating effect of self-efficacy on the indirect association between resilience and anxiety through positive coping among healthcare workers during the spread of the SARS-CoV-2 Delta variant since self-efficacy moderated the second stage of the mediation pathway. The result is in line with the Integrative Conceptual Framework of coping process (47) and previous studies (48, 49), reporting the impact of coping on health outcomes differs at different levels of self-efficacy. Practically speaking, healthcare workers with a higher level of general self-efficacy showed a stronger association between resilience and anxiety *via* positive coping. As shown in the Johnson-Neyman technique, the association between resilience and anxiety through positive coping was weakened with the decrease of general self-efficacy. Specifically, when the standard score of general self-efficacy dropped to below −0.8496, the indirect mediation effect was not significant any more. This result could be explained by the theory of self-efficacy developed by Bandura (77). General self-efficacy will determine whether an individual takes coping measures and how much an effort he or she will make. People with a higher sense of self-efficacy are more confident to face problems, while those with a lower sense of self-efficacy will avoid or follow the crowd rather than resisting pressure (78). Hence, healthcare workers with a lower sense of general self-efficacy are more likely to feel anxious even if they adopt a positive coping style.

### Implications

Our results have profound implications for the prevention of anxiety. The findings highlight the protective role of resilience and potential value of positive coping against anxiety among healthcare workers during the spread of the SARS-CoV-2 Delta variant. Programs combining resilience-building interventions [e.g., adopting a proactive orientation to solve problems, being flexible and adaptive (79)] and positive coping skills training [e.g., relaxation training, positive thinking, and problem solving (80)] should be designed and special attentions should be paid to healthcare workers with a higher sense of self-efficacy during the crisis.

### Limitations and Contributions

Some limitations should be recognized. First of all, this survey used a cross-sectional design, which leads to the inability to infer causality. Longitudinal studies could be carried out in the later study to further verify the moderated mediation model. Secondly, the cluster sampling method used in this study contributed to a high proportion of women compared to men. The reason for this phenomenon might be explained by the fact some medical positions, such as nurses, are mostly occupied by women, and other studies have shown similar limitations (71, 81). Thirdly, the information about occupation was not collected in our study, which might influence the results and the generalization of the findings. Fourthly, all data were collected through online self-report, which resulted in self-reported biases. Further study could collect information from multiple informants. Fifthly, all subjects came from a hospital in Jiangsu Province and there were only 390 subjects, which limited the generalization of the findings. Follow-up studies could recruit subjects from multiple hospitals in multiple provinces and cities. Finally, anxiety could be affected by numerous factors, the pathway identified in this study was just a part of them. Future studies could construct a more integrated model to explore the influential factors of anxiety.

As far as we know, this is the first study to assess the association between resilience and anxiety *via* positive coping among healthcare workers during the spread of the SARS-CoV-2 Delta variant, and to assess the moderating role of general self-efficacy, which would give insight into how resilience affects anxiety. From a practical point of view, this study plays an important role in maintaining the mental health of healthcare worker during the spread of the SARS-CoV-2 Delta variant.

## Conclusion

In summary, this study presented the protective effect of resilience on anxiety among healthcare workers during the spread of the SARS-CoV-2 Delta variant. Besides, positive coping could be one of the pathways through which resilience affects anxiety. Furthermore, the effect of resilience on anxiety *via* positive coping is enhanced with the increase of general self-efficacy.

## Data Availability Statement

The raw data supporting the conclusions of this article will be made available by the authors, without undue reservation.

## Ethics Statement

The studies involving human participants were reviewed and approved by Naval Medical University. The patients/participants provided their written informed consent to participate in this study.

## Author Contributions

YL and TH contributed to the writing of this article and the statistical analysis. WDo led the whole study including carrying out this study and putting forward the study. HG, JW, XS, YX, and WDe contributed to the data collection and statistical analysis. All authors contributed to editing the manuscript and have approved the final manuscript.

## Conflict of Interest

The authors declare that the research was conducted in the absence of any commercial or financial relationships that could be construed as a potential conflict of interest.

## Publisher's Note

All claims expressed in this article are solely those of the authors and do not necessarily represent those of their affiliated organizations, or those of the publisher, the editors and the reviewers. Any product that may be evaluated in this article, or claim that may be made by its manufacturer, is not guaranteed or endorsed by the publisher.
